# Antibiotic resistance among bacteria isolated from war-wounded patients at the Weapon Traumatology Training Center of the International Committee of the Red Cross from 2016 to 2019: a secondary analysis of WHONET surveillance data

**DOI:** 10.1186/s12879-022-07253-1

**Published:** 2022-03-14

**Authors:** Sally Yaacoub, Claudia Truppa, Thomas Ingemann Pedersen, Hicham Abdo, Rodolfo Rossi

**Affiliations:** 1 International Committee of the Red Cross (ICRC), Geneva, Switzerland; 2 Dar El Chifaa Hospital, Tripoli, Lebanon; 3 International Committee of the Red Cross (ICRC), Beirut, Lebanon

**Keywords:** War wounds, Wound infection, Bacterial drug resistance, Multidrug-resistance, Refugees, Vulnerable populations

## Abstract

**Background:**

A substantial body of evidence has recently emphasized the risks associated with antibiotic resistance (ABR) in conflicts in the Middle East. War-related, and more specifically weapon-related wounds can be an important breeding ground for multidrug resistant (MDR) organisms. However, the majority of available evidence comes from the military literature focused on risks and patterns of ABR in infections from combat-related injuries among military personnel. The overall aim of this study is to contribute to the scarce existing evidence on the burden of ABR among patients, including civilians with war-related wounds in the Middle East, in order to help inform the revision of empirical antibiotic prophylaxis and treatment protocols adopted in these settings. The primary objectives of this study are to: 1) describe the microbiology and the corresponding resistance profiles of the clinically relevant bacteria most commonly isolated from skin, soft tissue and bone biopsies in patients admitted to the WTTC; and 2) describe the association of the identified bacteria and corresponding resistance profiles with sociodemographic and specimen characteristics.

**Methods:**

We retrospectively evaluated the antibiograms of all consecutive, non-duplicate isolates from samples taken from patients admitted to the ICRC WTTC between 2016 and 2019, limited to skin and soft tissue samples and bone biopsies. We collected data on socio-demographic characteristics from patient files and data on specimens from the WHONET database. We ran univariate and multivariable logistic regression models to test the association between bacterial and resistance profiles with sociodemographic and specimen characteristics.

**Results:**

Patients who were admitted with war-related trauma to the ICRC reconstructive surgical project in Tripoli, Lebanon, from 2016 to 2019, presented with high proportion of MDR in the samples taken from skin and soft tissues and bones, particularly Enterobacterales (44.6%), MRSA (44.6%) and *P. aeruginosa* (7.6%). The multivariable analysis shows that the odds of MDR isolates were higher in Iraqi patients (compared to Syrian patients) and in Enterobacterales isolates (compared to *S. aureus* isolates).

**Conclusions:**

Our findings stress the importance of regularly screening patients who present with complex war-related injuries for colonization with MDR bacteria, and of ensuring an antibiotic-sensitivity testing-guided antimicrobial therapeutic approach.

**Supplementary Information:**

The online version contains supplementary material available at 10.1186/s12879-022-07253-1.

## Background

Antibiotic resistance (ABR) is a growing global health concern, with one of the major drivers of its emergence being inadequate antibiotic prescription at health system level and improper antibiotic use at population level [1–4].Antibiotic resistance (ABR) is a growing global health concern, with one of the major drivers of its emergence being inadequate antibiotic prescription at health system level and improper antibiotic use at population level [1–4].

In low- and middle-income countries (LMICs), the multifactorial origin of ABR is further compounded by additional contextual considerations, related to access to care, availability of drugs, and lack of health governance [5]. Poor or delayed access to care include having diagnostic difficulties, and suboptimal diagnostic capacities, that lead to inadequate antibiotic treatment [6]. However, access to care is not the only issue, but it is rather coupled with the quality of care, as access does not guarantee an adequate quality of care, and consequently adequate antibiotic treatment [7].

Despite the inequities in access to antibiotics in these contexts, their trends of consumption are rapidly reaching the same levels of high-income countries (HICs), increasing the potential for development of resistances [8].

LMICs are disproportionately more exposed to social and political instability compared to HICs. In fact, the 39 fragile and conflict-affected situations identified by the World Bank represent almost one third of all the countries classified as LMICs [9, 10]. Socio-political fragility and armed conflicts are strongly associated with poverty, and ABR can create additional financial burden on health care systems in these settings [11].

A substantial body of research conducted in particular in the Middle East has recently emphasized the risks associated with ABR in conflicts [12], identifying additional potential drivers contributing to its emergence and spread [13, 14].

War-related, and more specifically weapon-related wounds can be an important breeding ground for multidrug resistant (MDR) isolates. The military literature from the Middle East consistently highlights the risks and patterns of ABR in infections from combat-related injuries among military personnel [15–20]. However, there is scarce evidence on the risks and patterns of ABR among civilians wounded in conflict, and this evidence mainly comes from studies conducted by Médecins Sans Frontières (MSF) in Jordan. These studies report a concerning risk not only of ABR related to wounds, but also a high risk of ABR in osteomyelitis [21–23]. Studies conducted in Lebanon on Syrian civilians with weapon-related wounds have also reported worrying prevalence of infections, with increased risk of ABR in patients with delayed access to wound care and previous use of antibiotics without prior culture or antimicrobial susceptibility testing performed [24, 25].

From 2014 to 2021, the International Committee of the Red Cross (ICRC) ran a reconstructive war surgery center in Dar el Chifaa Hospital, Tripoli, in Lebanon—the Weapon Traumatology Training Center (WTTC). The WTTC provided highly specialized, multidisciplinary reconstructive and rehabilitative care for weapon-wounded patients in Lebanon. Its target population were patients residing in Lebanon, including Lebanese, Syrian, and Palestinian patients, and those referred from other ICRC projects in other countries of the region—mainly from Syria, Iraq and Yemen [26]. With the availability of microbiological data from the laboratory of Dar el Chifaa Hospital from 2016 to 2019, we analyzed the microbiological profiles and corresponding patterns of ABR among the samples retrieved from skin, soft tissue and bone biopsies performed on the patients hospitalized throughout this period of time.

## Objectives

The overall aim of this study is to contribute to the scarce existing evidence on the burden of ABR among patients with war-related wounds in the Middle East, in order to help inform the revision of empirical antibiotic prophylaxis and treatment protocols adopted in these settings.

The primary objectives of this study are to:

1. describe the microbiology and the corresponding resistance profiles of the clinically relevant bacteria most commonly isolated from skin, soft tissue and bone biopsies in patients admitted to the WTTC;
2. describe the association of the identified bacteria and corresponding resistance profiles with sociodemographic and specimen characteristics.

## Methods

### Setting

The WTTC was a project run by the ICRC from 2014 until the beginning of 2021, for the management of patients with weapon or war-related injuries. While initially established to respond to the acute surgical needs of patients wounded in Syria and seeking refuge in Lebanon, it rapidly evolved to adapt to the progressively evolving profile of patients admitted. In fact, the caseload of patients shifted from acute conflict-related trauma cases to cases with chronic complications of conflict-related trauma. The project evolved therefore from acute orthopedic surgery to highly specialized reconstructive surgical care. The admission criteria for the WTTC included both chronic orthopedic complications of war injuries (such as chronic osteomyelitis, mal-unions and non-unions), and chronic plastic and maxillo-facial complications from both traumas and burns. More details on the admission criteria are presented in an additional document [see Additional file 1: Appendix S1].

The vast majority of patients came to the attention of WTTC years after the original trauma, and often after having undergone multiple surgeries in their home countries without any proper clinical nor microbiological documentation available.

The majority of patients self-identified as civilians. To the purpose of this study, the information was not retrievable as it was not included in the patients’ files, in order to guarantee adequate protection of the patient, as per ICRC’s standards.

Based on the patients’ needs, the WTTC provided comprehensive care that involved medical and surgical treatment as appropriate, physical rehabilitation, mental health and psycho-social support and/or pain management consultations. Patients who had an indication for microbiological testing, specimens were collected. These specimens were analyzed in the laboratory of Dar Al Chifaa hospital in Tripoli, Lebanon, where the WTTC was based.

### Study design, population and eligibility

We retrospectively evaluated the antibiograms of all consecutive, non-duplicate isolates from samples taken from patients admitted to the ICRC WTTC between January 1, 2016 and December 31, 2019. The antibiograms were exported from the WHONET database generated by Dar Al Chifaa laboratory [27]. The WHONET database is a software package for the management of microbiology laboratory data and the analysis of antimicrobial susceptibility test results [28].

We included all patients admitted to WTTC with a specimen collected from bone, skin or soft tissue regardless of the type of injury or service/treatment provided. Unique cultures were defined as specimens for 1) different patients; 2) different organisms from the same patient; or 3) same organisms isolated from different sites of isolation for the same patient on different occasions separated by several days. If the same organism was isolated from the same patient on the same day of specimen collection, but from different specimen sites, it was only considered once, prioritizing bone over skin and soft tissue specimens. We did not have any exclusion criteria.

### Microbiological testing

The 2012 European Society of Clinical Microbiology and Infectious Diseases (ESCMID) Manual of Microbiology guideline was followed [29]. The antibiotic susceptibility was conducted based on the European Committee on Antimicrobial Susceptibility Testing (EUCAST) recommendations (available at the time of specimen collection), using the disk diffusion method [30]. MDR was defined as non-susceptibility to at least one agent in three or more antimicrobial categories, according to the European Centre for Disease Prevention and Control (ECDC) and the US Centers for Disease Control and Prevention (CDC) definitions [31]. MDR was assessed for the following bacteria: *Staphylococcus aureus*, Enterococci, Enterobacterales, *Pseudomonas aeruginosa* and *Acinetobacter baumannii*. In accordance with ECDC definitions*,* we considered Methicillin-resistant *Staphylococcus aureus* (MRSA) as MDR.

### Data collection and analyses

We collected data from patient files on socio-demographic characteristics including age, gender and nationality. The data on specimens was collected from the WHONET database, which included the specimen types, microorganisms, year of isolation and antibiograms. The antibiograms were exported in an excel file from the hospital’s WHONET database. The ICRC did not have direct access to the WHONET software, and therefore could not use the function of automatically generating the categories for drug resistance (multidrug-resistant, extensively drug-resistant and pandrug-resistant). The categorization of MDR was performed in IBM SPSS Statistics v.26 following the ECDC definitions of having resistance to at least one agent in three or more antimicrobial categories [31].

We reported resistance profiles for clinically relevant bacteria to the subject population. We reported descriptive data using percentages for categorical variables and medians with interquartile ranges (IQR) for continuous variables, since they had a non-normal distribution as tested through the Shapiro–Wilk test. We used the Chi-square test, Fisher’s exact test, Kruskal–Wallis test and Mann–Whitney U test, when applicable. We also conducted univariate and multivariable logistic regression models, to test the association between bacterial and resistance profiles with sociodemographic and specimen characteristics. We reported the unadjusted and adjusted odds ratios (ORs) with the 95% confidence intervals (CI). We considered a p-value < 0.1 as marginally significant and p-value < 0.05 as statistically significant. We used IBM SPSS Statistics v.26 for statistical analyses.

## Results

### Description of the patients and isolates

We identified 672 patients admitted to the WTTC who had at least one culture taken, with a total of 3204 cultures taken. Out of these cultures, 1149 (35.8%) yielded positive results. Approximately one third (30.3%, n = 348) were unique positive cultures from bone or skin and soft tissues from 198 patients admitted to the WTTC. The mean number of isolates per patient was 2.79 (standard deviation = 1.86 and range: 1–9). The median age of these patients (n = 198) was 33.5 years [IQR 25–45]. The majority were male patients (83.3%, n = 165) and were from Syria (75.3%, n = 149). Other patients were from Iraq (8.1%, n = 16), Lebanon (8.1%, n = 16), Palestine (5.6%, n = 11), and Yemen (3%, n = 6).

More than half of the 348 specimens were collected from skin and soft tissues (SST) (56.9%, n = 198), and the remaining from bone (43.1%, n = 150). The identified isolates from the 348 specimens included *S. aureus* (49.1%, n = 171), Enterobacterales (28.5%, n = 99), *P. aeruginosa* (13.2%, n = 46), Enterococci species (3.2%, n = 11) and *A. baumannii* (2%, n = 7). The identified bacteria are presented in Additional file 1. Appendix S2. The age of the patient was marginally associated with the identified bacteria (p-value < 0.049). There was no statistically significant association between the identified isolates and other sociodemographic characteristics and specimen characteristics (Table 1).

**Table 1:** Characteristics of the identified isolates from the specimens of bone and skin and soft tissues of patients with war-related injuries (N = 348)

Characteristic	*S. aureus* (N = 171) n (%)*	Enterobacterales (N = 99) n (%)*	*P. aeruginosa* (N = 46) n (%)*	Other bacteria^†^ (N = 32) n (%)*	p-value‡	Total (N = 348) n (%)^§^
Sociodemographic						
Age** [IQR], y	33 [25–43]	33 [25–48]	35 [26.5–45.3]	41.5 [33.3–50.3]	0.049	34.5 [26–44]
Sex					0.702	
Male	144 (50.5)	80 (28.1)	36 (12.6)	25 (8.8)		285 (81.9)
Female	27 (42.9)	19 (30.2)	10 (15.9)	7 (11.1)		63 (18.1)
Nationality					0.400	
Syria	138 (51.1)	71 (26.3)	37 (13.7)	24 (8.9)		270 (77.6)
Iraq	13 (48.1)	9 (33.3)	4 (14.8)	1 (3.7)		27 (7.8)
Lebanon	11 (50.0)	6 (27.3)	3 (13.6)	2 (9.1)		22 (6.3)
Palestine	5 (27.8)	7 (38.9)	2 (11.1)	4 (22.2)		18 (5.2)
Yemen	4 (36.4)	6 (54.5)	0 (0.0)	1 (9.1)		11 (3.2)
Specimen						
Site					0.844	
SST	101 (51.0)	55 (27.8)	24 (12.1)	18 (9.1)		198 (56.9)
Bone	70 (46.7)	44 (29.3)	22 (14.7)	14 (9.3)		150 (43.1)
Year of collection					0.498	
2016	36 (41.9)	32 (37.2)	9 (10.5)	9 (10.5)		86 (24.7)
2017	51 (52.0)	25 (25.5)	16 (16.3)	6 (6.1)		98 (28.2)
2018	40 (49.4)	24 (29.6)	9 (11.1)	8 (9.9)		81 (23.3)
2019	44 (53.0)	18 (21.7)	12 (14.5)	9 (10.8)		83 (23.8)

*IQR* Interquartile range, *SST* skin and soft tissue, *y* years

*The percentages are calculated based on the number of isolates reported under ‘Total’ per row

**Continuous variables are presented as medians. The Kruskal–Wallis test was used for the relevant statistical analysis

^†^Other bacteria include coagulase-negative staphylococci (n = 7), *Acinetobacter baumannii* (n = 7), Enterococcus species (n = 11), and *Streptococcus group A* (n = 7)

^‡^The Chi-square test or the Fisher’s exact test (when the expected cell counts are < 5) were used for the relevant statistical analysis, except for the variable age

^§^The percentages are calculated based on the total N = 348

### Resistance profiles

We identified 186 MDR isolates of the 334 isolates (55.7%) where we could apply the ECDC definitions of MDR (i.e., includes all isolates except coagulase-negative staphylococci and group A streptococci). For *S. aureus* isolates, the presence of MRSA was associated with the site of the isolate. The odds of MRSA were 2 times more likely in bone specimens compared to those in SST specimens (OR = 1.98 95% CI [1.07, 3.689], p-value = 0.029). Other sociodemographic and specimen characteristics were not associated with MRSA. In addition, more than 80% of the Enterobacterales were MDR (n = 83). Although the proportion of MDR was higher in male patients, patients from Syria and bone specimens, the differences were not statistically significant. For *P. aeruginosa*, almost one third of the isolates were considered MDR (30.4%, n = 14). However, the proportion of MDR *P. aeruginosa* did not differ based on sociodemographic (age, sex, and nationality) or specimen characteristics (site, and year). Additional information on the association of characteristics with each specific microorganism group is presented in Additional file 1. Appendix S3.

When combining all the MDR isolates, the proportion of MDR is highest in Enterobacterales (44.6%, n = 83) and *S. aureus* isolates (44.6%, n = 83), followed by *P. aeruginosa* isolates (7.6%, n = 14) (p-value < 0.001). The majority of MDR isolates are from patients from Syria (72.6%, n = 135). On the other hand, isolates from patients from Iraq had the highest proportion of MDR (85.2%, n = 23, N = 27). The multivariable analysis shows that MDR isolates are associated with patients from Iraq (p-value = 0.026). The odds of MDR isolates is 5.9 times higher in patients from Iraq compared to those from Syria (95% CI [1.84, 18.84]). In addition, MDR isolates were associated with the type of bacteria isolated (p-value < 0.001). The odds of MDR was 5.7 higher among Enterobacterales isolates compared to those among *S. aureus* isolates (95% CI [2.98, 10.76]). On the other hand, *P. aeruginosa* isolates were 57% less likely to be MDR compared to *S. aureus* isolates (adjusted OR = 0.43 95% CI [0.20, 0.89]). The odds of MDR were 59% higher in specimens from bone compared to those from SST (95% CI [0.96, 2.63], p-value = 0.071), which was marginally significant. There was no association between MDR isolates and age (p-value = 0.786), sex (p-value = 0.480) or year of specimen collection (p-value = 0.723). The detailed results are presented in Table 2.

**Table 2:** Factors associated with multi-drug resistant isolates identified from the specimens of bone and skin and soft tissues of patients with war-related injuries

Factor	MDR (N = 186) n (%)	Unadjusted OR (95% CI)*	p-value**	Adjusted OR (95% CI)^†^	p-value**
Sociodemographics					
Age	–	0.993 (0.978, 1.009)	0.389	0.998 (0.980, 1.016)	0.786
Sex					
Male	156 (83.9)	1	0.410	1	0.480
Female	30 (16.1)	0.789 (0.449, 1.386)		0.791 (0.412, 1.518)	
Nationality					
Syria	135 (72.6)	1	0.006	1	0.026
Iraq	23 (12.4)	5.28 (1.777, 15.699)		5.899 (1.848, 18.835)	
Lebanon	9 (4.8)	0.636 (0.263, 1.539)		0.624 (0.228, 1.712)	
Palestine	10 (5.4)	1.837 (0.611, 5.523)		1.597 (0.448, 5.698)	
Yemen	9 (4.8)	4.133 (0.876, 19.5)		1.964 (0.364, 10.612)	
Specimen					
Bacteria					
*S. aureus*	83 (44.6)	1	< 0.001	1	< 0.001
Enterobacterales	83 (44.6)	5.5 (2.978, 10.15)		5.662 (2.981, 10.755)	
*P. aeruginosa*	14 (7.6)	0.464 (0.231, 0.93)		0.427 (0.204, 0.893)	
*A. baumannii*	3 (1.6)	0.795 (0.173, 3.66)		0.801 (0.163, 3.941)	
Enterococci	3 (1.6)	0.398 (0.102, 1.55)		0.361 (0.085, 1.532)	
Site					
SST	97 (52.2)	1	0.051	1	0.071
Bone	89 (47.8)	1.551 (0.999, 2.410)		1.590 (0.961, 2.632)	
Year of collection					
2016	46 (24.7)	1	0.432	1	0.723
2017	56 (30.1)	1.187 (0.659, 2.319)		1.181 (0.59, 2.367)	
2018	47 (25.3)	1.328 (0.710, 2.484)		1.18 (0.564, 2.467)	
2019	37 (19.9)	0.804 (0.433, 1.495)		0.819 (0.400, 1.678)	

*CI* confidence interval, *MDR* multi-drug resistant, *OR* odds ratio, *SST* skin and soft tissue

*Univariate logistic regression models were conducted

**p-value from Likelihood Ratio Test

^†^Multivariable logistic regression model was conducted

We report below the antimicrobial susceptibility tests of the clinically relevant isolates.

#### Staphylococcus aureus

**Fig. 1:**
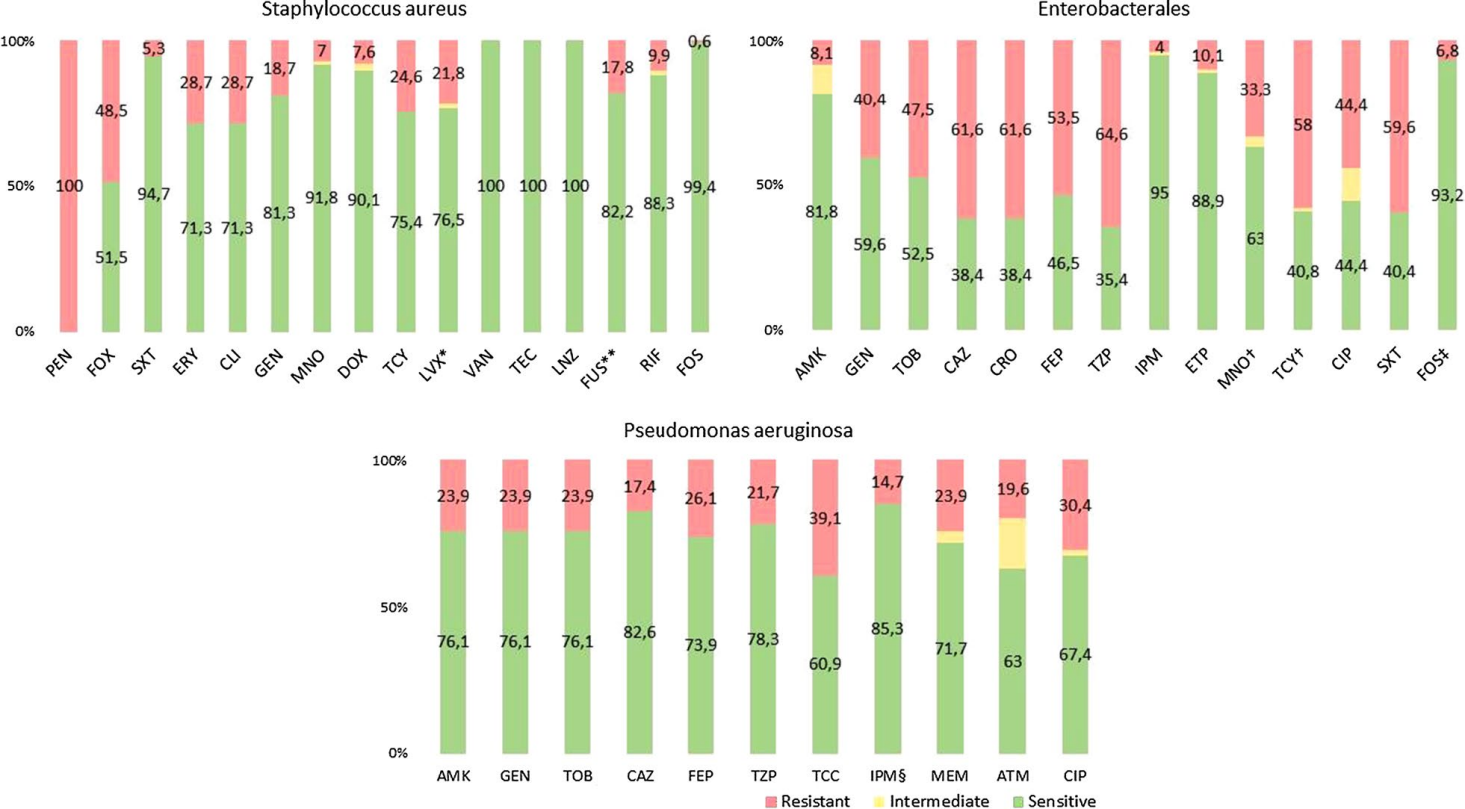
Antibiotic resistance profiles of *Staphylococcus aureus* (n = 171), Enterobacterales (n = 99), and *Pseudomonas aeruginosa* isolates (n = 46). *n = 119, **n = 157, ^†^n = 81, ^‡^n = 74, ^§^n = 34. *AMK* Amikacin, *ATM* Aztreonam, *CAZ* Ceftazidime, *CIP* Ciprofloxacin, *CLI* Clindamycin, *CRO* Ceftriaxone, *DOX* Doxycycline, *ERY* Erythromycin, *ETP* Ertapenem, *FEP* Cefepime, *FOS* Fosfomycin, *FOX* Cefoxitin, *FUS* Fusidic acid, *GEN* Gentamicin, *IPM* Imipenem, LNZ: Linezolid, *LVX* Levofloxacin, *MEM* Meropenem, *MNO* Minocycline, *PEN* Penicillin, *SXT* Trimethoprim-sulfamethoxazole, *TCC* Ticarcillin-clavulanic acid, *TCY* Tetracycline, *TEC* Teicoplanin, *TOB* Tobramycin, *TZP* Piperacillin-tazobactam, *VAN* Vancomycin

All identified *S. aureus* were resistant to penicillin, and none were resistant to linezolid or vancomycin. Almost half of the isolates were resistant to cefoxitin, (i.e., MRSA) (48.5%, n = 83). Additionally, the majority of the isolates were susceptible to trimethoprim-sulfamethoxazole with only 5.3% resistant (n = 9). Almost a third of the isolates were resistant to clindamycin (28.6%, n = 49) and 18.7% (n = 32) resistant to gentamicin. As for the tetracyclines, 7.6% were resistant to doxycycline (n = 13). The detailed antibiotic resistance profiles are presented in Fig. 1.

#### Enterobacterales

We identified 99 specimens with Enterobacterales isolates including *Enterobacter cloacae, Escherichia coli* (*E. coli*)*, Proteus mirabilis, Klebsiella pneumoniae, Morganella morganii, Citrobacter freundii* and *Serratia marcescens*. Regarding the susceptibility to aminoglycosides, 8% of the isolates were resistant to amikacin (n = 8) and 40% resistant to gentamicin (n = 40). More than half were resistant to the 3^rd^ and 4^th^ generation cephalosporins ceftazidime (61.6%, n = 61), ceftriaxone (61.6%, n = 61) and cefepime (53.5%, n = 53). Similarly, for the piperacillin-tazobactam where 64 isolates were resistant (64.7%). The majority of the isolates were susceptible to imipenem and ertapenem with only four (4%) and ten (10.1%) resistant isolates, respectively. Out of the isolates where the susceptibility of tetracyclines was assessed (n = 81), one third was resistant to minocycline (33.3%, n = 27) and 58% resistant to tetracycline (n = 47). Additionally, 44.4% of the isolates were resistant to ciprofloxacin (n = 44), and 59.6% were resistant to trimethoprim-sulfamethoxazole (n = 59). The detailed antibiotic resistance profiles are presented in Fig. 1 for Enterobacterales and in Additional file 1. Appendix S4 for each bacteria.

#### Pseudomonas aeruginosa

*P. aeruginosa* was isolated from 46 specimens. Of those, 11 were resistant to gentamicin (23.9%), and a similar proportion of resistant isolates were identified for the other aminoglycosides, amikacin and tobramycin. Approximately 17.5% (n = 8) were resistant to ceftazidime, and 26.1% (n = 12) resistant to cefepime. In addition, 21.7% (n = 10) were resistant to piperacillin-tazobactam. Regarding carbapenems, almost one quarter were resistant to meropenem (23.9%, n = 11) and 15% were resistant to imipenem (5 out of 34 tested specimens). In addition, 20% were resistant to aztreonam (n = 9) and 30% to ciprofloxacin (n = 14). The detailed antimicrobial susceptibility test statistics are presented in Fig. 1.

#### Other bacteria

Out of the total 11 Enterococci isolated, three were resistant to levofloxacin, eight to gentamicin, six to streptomycin and one to ampicillin. None of the isolates were resistant to tigecycline, linezolid, vancomycin, or teicoplanin. Three of the 11 Enterococci isolates were considered MDR.

*Acinetobacter baumannii* was isolated from seven specimens. Three isolates were resistant to gentamicin and other aminoglycosides. In addition, three isolates were resistant to ceftazidime, piperacillin-tazobactam, imipenem, meropenem and ciprofloxacin. Out of the seven isolates, two were resistant to trimethoprim-sulfamethoxazole. For tetracyclines, 3 (out of 6 isolates) were resistant to doxycycline and none were resistant to minocycline. Finally, three of the seven *A. baumannii* isolates were considered MDR.

## Discussion

### Summary and interpretation of findings

In our study, we were able to identify the microbiological profiles and patterns of ABR of isolates from weapon-wounded civilians. The most commonly isolated bacteria was *S. aureus* (49.1%), followed by Enterobacterales (28.5%), *P. aeruginosa* (13.2%), Enterococci species (3.2%) and *A. baumannii* (2%). Our findings are similar to those reported by Fily et al. where *S. aureus* was also the most frequently isolated bacteria, with similar proportions of *Enterobacteriaceae* (31.5%), *P. aeruginosa* (13.5%) and *A. baumannii* (2.8%) [32]. However, the proportion of *S. aureus* reported by Fily et al. (26.3%) was lower than that in our findings [32]. One explanation may be the inclusion of only bone samples from patients with osteomyelitis with the exclusion of soft tissue samples, in contrast to our study, which included SST samples (including superficial swabs), regardless of the underlying infection.

When comparing our results to that from military personnel, the microbiological profiles of confirmed extremity wound infections of military personnel were different with proportions of *S. aureus* isolates of 3% and *A. baumannii* of 17% [19]. In fact, one study on patients admitted to a military hospital in Iraq showed statistically significant differences between U.S. military patients and non-military non-U.S. patients [33]. The isolated bacteria of U.S. military patients compared to non-military non-U.S. patients included *S. aureus* (26 vs. 5%), *K. pneumoniae* (3 vs. 13%), and *P. aeruginosa* (3 vs. 10%) [33]. The differences in profiles among isolates from military personnel as compared to civilians might be due to several factors, including—although not limited to—the timeliness and quality of care they have access to at the moment of injury.

When comparing our results to similar studies conducted among civilian weapon wounded, the proportion of MDR isolates reported in our study is lower than that reported by MSF where the same definitions of MDR are applied. The study by Alga et al. reports that MDR was detected in 73% of patients with positive wound cultures resulting from conflict-related injuries (versus 55.7% from our study) [34]. Other small studies among civilian patients also report a higher proportion of MDR with 69% MDR isolates from war-associated wound infections [23] and 66% MDR isolates from post-trauma infections [35]. In addition, the proportion of MDR is still higher in other studies compared to our study, even when we solely consider specimens from bone cultures (61.8%). Possible explanations for the discrepancy can be that other studies included only patients with clinical signs of an infection [23, 34], only infections of acute injuries [34], a small sample [23, 34, 35] and/or different definitions of MDR [23, 35]. On the other hand, the isolates from confirmed extremity wound infections of military personnel had lower proportion of MDR ranging between 32 and 44% [19].

Our results show higher odds and proportion of MDR amongst Enterobacterales. This is similar to the available literature on MDR Enterobacterales, with a proportion of MDR ranging between 63% for Proteus and 100% for *E. coli* [34]. These proportions were higher than that of other isolates reported in the same study (e.g., MRSA and *P. aeruginosa*) [34]. We identified that isolates from patients from Iraq had higher odds of MDR compared to that from other countries. One possible explanation might be the high proportion of Enterobacterales amongst this group of patients. Other possible explanations can be that Iraqi patients had longer delay since injury, a greater number of previous surgeries before presenting to WTTC, more antibiotic treatment courses, presence of polymicrobial infections [32] and/or high community resistance rates [36].

Our results on the proportion of MRSA (48.5%) are consistent with the literature as a systematic review by Truppa et al. report a percentage median resistance in conflict-affected countries of 43.37% [30]. Another study reported 42% MRSA among *S. aureus* isolates in Syrian patients with war-associated wound infections [23]. Likewise, for the proportion of MDR Enterobacterales (83.8%), Fily et al. report a similar proportion of MDR Enterobacteriaceae (86.2%), although the latter only includes isolates from post-traumatic osteomyelitis [32]. Evidence of MDR Enterobacterales in the Middle East region suggest that it is endemic for carbapenemase-producing Enterobacterales [39, 40]. In addition, the lowest proportion of isolates was *A. baumannii* isolates (n = 7, 2%). This is comparable to another study on chronic osteomyelitis due to war injury where the proportion of *A. baumannii* isolates was also the lowest among the different isolates (n = 6, 4%) [21]. Fily et al. also reported a similar proportion of *A. baumannii* isolates (n = 21, 2.8%). Additionally, MDR *A. baumannii* isolates have been reported in war injuries [21, 41–43]. In our study, three out of seven *A. baumannii* isolates were MDR. Murphy et al. also reported a similar proportion of MDR *A. baumannii* isolates (three out of six) in Iraqi civilians with war-related chronic osteomyelitis [21].

### Strengths and limitations of the study

This study has a number of strengths. We have reported the susceptibility of bacteria isolated from war-wounded civilians, adding to the literature on a specific population for which there is limited available literature. We also did not restrict our inclusion to particular bacteria, rather we included all isolated bacteria from the population of interest. In addition, we used data from the WHONET database, a uniform standardized database. This ensured the homogeneity of the data and allowed the comparison of the microbiological susceptibility data of different years and of that reported in different studies in the literature.

On the other hand, there are several limitations to this study. One limitation is the retrospective design of the study based on laboratory data. There was missing and/or limited information on the clinical presentation, medical history and sociodemographic characteristics of the patients. It was not possible to discern between different stages across the continuum of wound infections, namely: contamination, defined as presence or proliferation of bacteria without any sign of local or systemic inflammation; critical colonization (defined as presence of microbiological isolates without signs of inflammation but interfering with the process of wound healing); and infection (i.e., skin and soft tissue infections, bone infections, prosthetic infections, or concomitant infections) [44, 45]. Another limitation is the small sample size or possible confounding because of which we might not have been able to detect statistically significant associations, as in the case of the association between the specimen type and MDR, which was only marginally significant. An important additional limitation of this study lies with the lack of possibility to discriminate before community *versus* hospital-acquired infections, as the date of admission of the patient and collection of the samples could not be used as proxy measure for the timing of the colonization/infection. In fact, the vast majority of patients were affected by chronic complications of war-related wounds. Because of this, they came to the attention of the ICRC after an important clinical journey which implied previous outpatient and/or inpatient care, for which documentation was often not available to the ICRC care providers; on the other hand, the timing of sample collection, particularly for bone biopsies, but also for superficial wound, would often be deferred beyond the first 24–48 h from admission, in order to proceed with the complete clinical workout needed in preparation for the elective surgery. Finally, although the WHONET is a standardized method of reporting microbiological data, due to the poor harmonization and low standardization of surveillance of ABR in the Middle East, it is difficult to compare data from other studies conducted on war-wounded civilians in this setting [38].

### Implications for clinical practice and future research

Based on the findings of our study, we propose strengthening antibiotic stewardship in general, and in orthopedic surgical projects conducted in similar settings, in specific. This is of great importance especially that antimicrobial stewardship programs in health care facilities have shown a positive impact in LMICs [46]. We also reinforce the recommendation already formulated by MSF that bone biopsies be regularly conducted before reconstructive orthopedic surgical interventions in weapon-wounded civilians in such settings [32, 34].

The ICRC guidelines for antibiotic prophylaxis and treatment in war wounds were first published in 2010 [47] and revised in 2019 [48]. These are based on recommendations by WHO [49], MSF (personal communication following consultations with the MSF team in their Amman reconstructive surgical project), army guidelines and review papers, and provide the basis of war wounds management for many international organizations offering surgical services in conflict-affected settings.

The specific ICRC guidelines for antibiotic prophylaxis in elective surgery in the context of the WTTC project were adapted from existing American [50], Scottish [51] and Swiss guidelines [52] for reconstructive orthopedic surgery. These guidelines restrict the use of antibiotic prophylaxis in orthopedic surgery to cases with implant insertion. A single dose of cefazolin is recommended, unless there is evidence that the patient harbors bacteria warranting use of a different antibiotic prophylaxis (e.g. MRSA colonization/infection, other MDR bacteria). In cases of acute orthopedic surgery, e.g. in the acute weapon-wounded, cefazolin is also the recommended first-line antibiotic, but with the addition of gentamicin and metronidazole, if the injury is more than 72 h old [48]. In these cases, antibiotics are given for 48 to 72 h. The addition of gentamicin is suggested in the presence of signs of local inflammation, while that of metronidazole roots in the knowledge that the risk of anaerobic infections increases with time from injury to delayed surgery, and acknowledging the difficulties to culture anaerobic bacteria even under optimal circumstances [48].

Where empirical treatment of SST and bone infections is warranted, whether pending results of cultures or due to lack of microbiological diagnostics, cefazolin is also first choice. In septic patients, the empirical treatment regimen is a combination of cefazolin, gentamicin and metronidazole. However, if there is no adequate response within few days, then the treatment options adopted in the management of complicated war wounds in WTTC were the switch to meropenem, with the potential addition of vancomycin in case the patient was still not adequately responding. These guidelines were mostly based on the surgeons’ previous experience in treating similar cases in different settings, as well as on the high prevalence of ESBL and MRSA in the specific context of WTTC. The empirical treatment guidelines were used exclusively in cases where no microbiological evidence was available. In other cases, susceptibility profile-guided antibiotic prescription was the norm, under the guidance of the hospital infectious diseases specialist.

Considering the resistance profiles documented in the reconstructive surgical project implemented in the WTTC and reported in this study, an update of the current guidelines might be warranted. Moreover, when planning the implementation of a complex surgical project targeting patients presenting complex war wounds, a rigorous antibiotic stewardship protocol should be put in place, including the update of antibiotic prophylaxis and treatment protocols based on the continuous monitoring of the local resistance profiles, as it has already been stressed in other Middle Eastern settings [34].

Additionally, on a broader scale, there is a need to establish a robust national surveillance system in order to understand local resistance profiles, that should guide national guidelines for the management of infectious diseases. It would also be essential to adopt novel solutions for ABR testing, such as innovative accessible laboratories as the Mini-Lab designed by MSF [53], in order to expand the capacity of ABR testing, and ultimately reporting.

Finally, there is a need for large-scale prospective studies that consider the clinical presentation and medical history of patients when identifying resistance profiles and factors associated with resistance in a war-affected populations, as this would provide better insight on both the source of infection (community *versus* hospital-acquired), and therefore on the prophylactic and empirical antibiotic treatment protocols for civilians and military personnel.

## Conclusions

Patients who were admitted with war-related trauma to the ICRC reconstructive surgical project in Tripoli, Lebanon, from 2016 to 2019, presented with high proportion of MDR in the samples taken from skin and soft tissues and bones, particularly Enterobacterales (83.8%), MRSA (48.5%) and *P. aeruginosa* (30.4%). These findings stress the importance of regularly screening patients who present with complex war-related injuries for colonization with MDR bacteria, and of ensuring an antibiotic-sensitivity testing-guided antimicrobial therapeutic approach. Large scale prospective cohort studies among weapon wounded patients in the Middle Eastern region are needed in order to confirm these findings and ensure revision of the currently adopted clinical guidelines for antibiotic prophylaxis in orthopedic surgery, as well as epidemiologically driven clinical treatment protocols.

## Supplementary Information

**Additional file 1:** **Appendix S1. **Patient admission criteria to the International Committee of the Red Cross Weapon Traumatology Training Centre in Tripoli, Lebanon**. Appendix S2.** Bacteria identified from the specimens of bone and skin and soft tissues of patients with war-related injuries (N=348)**. Appendix S3.** Characteristics of the multi-drug resistant isolates of *Staphylococcus aureus*, Enterobacterales and *Pseudomonas aeruginosa *isolates.** Appendix S4.** Antibiotic resistance profiles of Enterobacterales isolates per bacteria.

## Data Availability

The datasets generated and/or analysed during the current study are not publicly available due confidentiality of the data of ICRC, but are available from the corresponding author on reasonable request.

## Abbreviations

ABR: Antibiotic resistance
AMK: Amikacin
ATM: Aztreonam
CAZ: Ceftazidime
CDC: Centers for Disease Control and Prevention
CI: Confidence interval
CIP: Ciprofloxacin
CLI: Clindamycin
CRO: Ceftriaxone
DOX: Doxycycline
ECDC: European Centre for Disease Prevention and Control
ERY: Erythromycin
ESCMID: European Society of Clinical Microbiology and Infectious Diseases
ETP: Ertapenem
EUCAST: European Committee on Antimicrobial Susceptibility Testing
FEP: Cefepime
FOS: Fosfomycin
FOX: Cefoxitin
FUS: Fusidic acid
GEN: Gentamicin
HICs: High-income countries
ICRC: International Committee of the Red Cross
IPM: Imipenem
IQR: Interquartile range
LMICs: Low- and Middle-Income Countries
LNZ: Linezolid
LVX: Levofloxacin
MDR: Multi-drug resistant
MEM: Meropenem
MNO: Minocycline
MRSA: Methicillin- resistant *Staphylococcus aureus*
MSF: Médecins sans frontières
OR: Odds ratio
PEN: Penicillin
SST: Skin and soft tissue
SXT: Trimethoprim-sulfamethoxazole
TCC: Ticarcillin-clavulanic acid
TCY: Tetracycline
TEC: Teicoplanin
TOB: Tobramycin
TZP: Piperacillin-tazobactam
VAN: Vancomycin
WTTC: Weapon Traumatology Training Center
