# Supplementary material for: Antibiotic resistance among bacteria isolated from war-wounded patients at the Weapon Traumatology Training Center of the International Committee of the Red Cross from 2016 to 2019: a secondary analysis of WHONET surveillance data
Source: BMC Infect Dis. 2022 Mar 14;22:257. doi: 10.1186/s12879-022-07253-1 (PMC8922823; doi:10.1186/s12879-022-07253-1)
Supplement: Supplementary file 1 — Additional file 1: Appendix S1. Patient admission criteria to the International Committee of the Red Cross Weapon Traumatology Training Centre in Tripoli, Lebanon. Appendix S2. Bacteria identified from the specimens of bone and skin and soft tissues of patients with war-related injuries (N=348). Appendix S3. Characteristics of the multi-drug resistant isolates of Staphylococcus aureus, Enterobacterales and Pseudomonas aeruginosa isolates. Appendix S4. Antibiotic resistance profiles of Enterobacterales isolates per bacteria. [file 12879_2022_7253_MOESM1_ESM.docx]

**ADDITIONAL FILE 1.**

**Appendix 1.** Patient admission criteria to the International Committee of the Red Cross Weapon Traumatology Training Centre in Tripoli, Lebanon

**Appendix 2.** Bacteria identified from the specimens of bone and skin and soft tissues of patients with war-related injuries (N=348)

**Appendix 3.** Characteristics of the multi-drug resistant isolates of *Staphylococcus aureus*, Enterobacterales and *Pseudomonas aeruginosa* isolates.

**Appendix 4.** Antibiotic resistance profiles of Enterobacterales isolates per bacteria

**Appendix 1.** Patient admission criteria to the International Committee of the Red Cross Weapon Traumatology Training Centre in Tripoli, Lebanon

Patient admission criteria to the International Committee of the Red Cross Weapon Traumatology Training Centre in Tripoli, Lebanon

The criteria for admission to the WTTC are divided into general and specific criteria as follows:

- General criteria:
- Non-acute war injuries with a focus on firearms and/or explosive devices, or related collateral damage:
  1. Chronic osteomyelitis
  2. Mal-unions
  3. Non-unions
  4. Soft tissue contractures
  5. Chronic wounds
  6. Nerve injuries which can recover functionality with secondary repair or tendon transfer
  7. Patients in need of physiotherapy or a service from the Physical Rehabilitation Programme (PRP) only

These injuries must have occurred after the date of conflict in the country of origin.

- Acute war injuries with a focus on firearms and/or explosive devices, or related collateral damages based on bed availability
- Specific criteria for the weapon-wounded (WW):

1. Orthopaedic:
   1. Major joint injuries requiring total joint replacement (hip, knee, shoulder)
   2. Ligamentous reconstruction for chronic joint instability (knee, wrist)
   3. Joint injuries requiring arthrodesis
   4. Injuries requiring elective amputations or stump revisions
   5. Re- amputations or stump revision as requested by the PRP (also non-WW patients)
   6. Hand injuries amenable to significant functional improvement with surgery
2. Plastic and maxillo-facial:
   1. Soft tissue defects requiring grafting, or pedicled flaps
   2. Burn contractures affecting the function
   3. Infected and non-infected, mal- or non-union with or without bone gap of facial bones (focus: reconstruction of mandibular and/or maxillary defects)
   4. Temporo-mandibular joint problems affecting mouth opening.
   5. Orbital injuries affecting vision
   6. Craniofacial injuries with no significant neural involvement
   7. Selected patients with chronic sequelae of burns
   8. Selected nerve injuries of the upper and lower limb

**Appendix 2.** Bacteria identified from the specimens of bone and skin and soft tissues of patients with war-related injuries (N=348)

**Appendix 3.** Characteristics of the multi-drug resistant isolates of *Staphylococcus aureus*, Enterobacterales and *Pseudomonas aeruginosa* isolates compared to non-multi-drug resistant isolates.

| **Characteristic*** | | **MRSA**  (N=83)  n (%) | **p-value** | **MDR**  **Enterobacterales**  (N=83)  n (%) | **p-value** | **MDR *P. aeruginosa***  (N=14)  n (%) | **p-value** |
| --- | --- | --- | --- | --- | --- | --- | --- |
| ***Sociodemographic*** | |  |  |  |  |  |  |
| **Age**** (IQR), y | | 32 [24-43] | 0.473 | 33 [25-48] | 0.591 | 35 [19-51] | 0.933 |
| **Sex** | |  |  |  |  |  |  |
|  | Male | 71 (85.5) | 0.643 | 68 (81.9) | 0.521 | 12 (85.7) | 0.423 |
|  | Female | 12 (14.5) |  | 15 (18.1) |  | 2 (14.3) |  |
| **Nationality** | |  |  |  |  |  |  |
|  | Syria | 62 (74.7) | 0.561 | 59 (71.1) | 0.502 | 9 (64.3) | 0.990 |
|  | Iraq | 9 (10.8) |  | 9 (10.8) |  | 4 (28.6) |  |
|  | Lebanon | 5 (6.0) |  | 3 (3.6) |  | 1 (7.1) |  |
|  | Palestine | 3 (3.6) |  | 7 (8.4) |  | 0 (0.0) |  |
|  | Yemen | 4 (4.8) |  | 5 (6.0) |  | 0 (0.0) |  |
| ***Specimen*** | |  |  |  |  |  |  |
| **Site** | |  | 0.029 |  | 0.951 |  | 0.403 |
|  | SST | 42 (50.6) |  | 46 (55.4) |  | 6 (42.9) |  |
|  | Bone | 41 (49.4) |  | 37 (44.6) |  | 8 (57.1) |  |
| **Year of collection** | |  | 0.635 |  | 0.263 |  | 0.222 |
|  | 2016 | 15 (21.1) |  | 25 (32.3) |  | 2 (14.3) |  |
|  | 2017 | 28 (29.8) |  | 19 (25.3) |  | 7 (50.0) |  |
|  | 2018 | 20 (23.4) |  | 23 (24.2) |  | 4 (28.6) |  |
|  | 2019 | 20 (25.7) |  | 16 (18.2) |  | 1 (7.1) |  |

Abbreviations: IQR: interquartile range, P*. aeruginosa*: *Pseudomonas* aeruginosa, *S. aureus: Staphylococcus aureus*, SST: skin and soft tissue, y: years.

*The Chi-square test or the Fisher’s exact test (when the expected cell counts are <5) were used for the relevant statistical analysis, except for the variable age.

**Continuous variables are presented as medians. The Mann-Whitney U test was used for the relevant statistical analysis.

**Appendix 4.** Antibiotic resistance profiles of Enterobactesrales isolates per bacteria

| **Bacteria** | **Resistant** (%) | | | | | | | | | | | | | | **MDR** (%) |
| --- | --- | --- | --- | --- | --- | --- | --- | --- | --- | --- | --- | --- | --- | --- | --- |
|  | AMK | GEN | TOB | CAZ | CRO | FEP | TZP | IPM | ETP | MNO* | TCY* | CIP | SXT | FOS** |  |
| ***Enterobacter cloacae* (n=25)** | 4 | 24 | 32 | 52 | 52 | 44 | 52 | 8 | 16 | 20 | 40 | 20 | 52 | 0 | 100 |
| ***Escherichia coli***  **(n=25)** | 0 | 40 | 48 | 76 | 76 | 64 | 80 | 0 | 0 | 16 | 60 | 68 | 56 | 10 | 88 |
| ***Proteus mirabilis***  **(n=22)** | 9 | 64 | 54 | 59 | 59 | 59 | 64 | 0 | 0 | 100 | 100 | 41 | 77 | 13 | 68 |
| ***Klebsiella pneumoniae* (n=18)** | 17 | 28 | 56 | 72 | 72 | 56 | 78 | 11 | 33 | 29 | 53 | 56 | 56 | 0 | 83 |
| **Enterobacterales (n=99)**† | 8 | 40 | 47 | 62 | 62 | 54 | 65 | 4 | 10 | 33 | 58 | 44 | 60 | 7 | 84 |

Abbreviations: AMK: Amikacin, CAZ: Ceftazidime, CIP: Ciprofloxacin, CRO: Ceftriaxone, ETP: Ertapenem, FEP: Cefepime, FOS: Fosfomycin, GEN: Gentamicin, I: Intermediate, IPM: Imipenem, MDR: multi-drug resistant, MNO: Minocycline, R: Resistant, SXT: Trimethoprim-sulfamethoxazole, TCY: Tetracycline, TOB: Tobramycin, TZP: Piperacillin-tazobactam.

*: the percentage is calculated based on the following n: *Proteus mirabilis* n=7, *Klebsiella pneumonia* n=17, Enterobacterales n=81

**: the percentage is calculated based on the following n: *Enterobacter cloacae* n=21, *Escherichia coli* n=20, *Proteus mirabilis* n=15, *Klebsiella pneumonia* n=14, Enterobacterales n=74

†: Enterobacterales also include *Morganella morganii* (n=6), *Citrobacter freundii* (n=2), *Serratia marcescens* (n=1)
